# Prospective Evaluation of Sleep Apnea as Manifestation of Heart Failure in Children

**DOI:** 10.1007/s00246-015-1269-3

**Published:** 2015-10-16

**Authors:** Susanna L. den Boer, Koen F. M. Joosten, Sandra van den Berg, Ad P. C. M. Backx, Ronald B. Tanke, Gideon J. du Marchie Sarvaas, Willem A. Helbing, Lukas A. J. Rammeloo, Arend D. J. ten Harkel, Gabriëlle G. van Iperen, Michiel Dalinghaus

**Affiliations:** Department of Pediatrics, Division of Pediatric Cardiology, Sophia Children’s Hospital, Erasmus Medical Center, Rotterdam, Dr. Molewaterplein 60, Room Sp-2433, 3000 CB Rotterdam, The Netherlands; Department of Pediatrics, Pediatric Intensive Care, Sophia Children’s Hospital, Erasmus Medical Center, Rotterdam, The Netherlands; Department of Pediatrics, Division of Pediatric Cardiology, Emma Children’s Hospital, Academic Medical Center, Amsterdam, The Netherlands; Department of Pediatrics, Division of Pediatric Cardiology, Radboud University Medical Center, Nijmegen, The Netherlands; Department of Pediatrics, Division of Pediatric Cardiology, Beatrix Children’s Hospital, University Medical Center Groningen, Groningen, The Netherlands; Department of Pediatrics, Division of Pediatric Cardiology, Free University Medical Center, Amsterdam, The Netherlands; Department of Pediatrics, Division of Pediatric Cardiology, Leiden University Medical Center, Leiden, The Netherlands; Department of Pediatrics, Division of Pediatric Cardiology, Wilhelmina Children’s Hospital, University Medical Center Utrecht, Utrecht, The Netherlands

**Keywords:** Dilated cardiomyopathy, Pediatric, Polysomnography, Central sleep apnea

## Abstract

In adults with heart failure, central sleep apnea (CSA), often manifested as Cheyne–Stokes respiration, is common, and has been associated with adverse outcome. Heart failure in children is commonly caused by dilated cardiomyopathy (DCM). It is unknown whether children with heart failure secondary to DCM have CSA, and whether CSA is related to the severity of heart failure. In this prospective observational study, 37 patients (<18 year) with heart failure secondary to DCM were included. They underwent polysomnography, clinical and laboratory evaluation and echocardiographic assessment. After a median follow-up time of 2 years, eight patients underwent heart transplantation. CSA (apnea–hypopnea index [AHI] ≥1) was found in 19 % of the patients. AHI ranged from 1.2 to 4.5/h. The occurrence of CSA was not related to the severity of heart failure. Three older patients showed a breathing pattern mimicking Cheyne–Stokes respiration, two of whom required heart transplantation. CSA was found in 19 % of the children with heart failure secondary to DCM. No relation was found with the severity of heart failure. In a small subset of children with severe DCM, a pattern mimicking Cheyne–Stokes respiration was registered.

## Introduction

In adults with heart failure, central sleep apnea (CSA) is highly prevalent [1–4]. Cheyne–Stokes respiration is a form of CSA and, in adults with heart failure, used as a synonym for CSA [5]. The occurrence of CSA in adults with heart failure is associated with the severity of heart failure and with higher mortality rates [1, 2, 6, 7].

Dilated cardiomyopathy (DCM) in children is a severe cardiac disorder resulting in heart failure. To the best of our knowledge, so far, no study has been published that has investigated whether CSA occurs in children with heart failure. According to the prevalence in adults, we speculated that CSA occurs in children with heart failure and may also be related to the severity of heart failure. Therefore, we conducted a prospective study to determine the prevalence of CSA and its clinical relevance in children with heart failure secondary to DCM.

## Methods

### Patient Selection

Between October 2010 and October 2013, children (<18 year) with DCM were asked to participate in a nationwide prospective follow-up study. DCM was defined as a left ventricle end-diastolic dimension (LVEDD) >95th percentile for body surface area and a shortening fraction (SF) ≤25 %. DCM was of idiopathic origin or secondary to other causes. Patients with DCM secondary to neuromuscular diseases were excluded, because sleep-disordered breathing can be present as result of muscular weakness [8, 9].

As part of the follow-up study, an overnight polysomnography was planned. Furthermore, a detailed clinical evaluation, including echocardiography, NT-pro BNP measurement and clinical assessment using the New York University Pediatric Heart Failure Index [10], was performed within 3 months of the polysomnography. Medication use and demographic data (age, gender and duration of DCM) were recorded. Follow-up data were collected through January 2015. Primary end points were death and heart transplantation. The review board of all participating centers approved the protocol. All parents, and patients ≥12 year, gave their written informed consent.

### Sleep Study

Patients underwent overnight polysomnography either at home or in hospital. Measurements at home were done with the Embletta Portable Diagnostic System and analyzed using Somnologica for Embletta Software 3.3 ENU (Medcare Flage, Reykjavik, Iceland). Embletta is a multichannel test that continuously measures respiration by a pressure transducer attached to a nasal cannula (Salter labs, Arvin USA), breathing effort through respiratory elastic belts at abdominal and chest level (X act), and oxygen saturation (SaO_2_) and heart rate using an infant or pediatric oxygen sensor (OxiMax; Nellcor, Pleasanton, USA) on a fingertip. Caregivers were instructed to apply all sensors and to start the measurement at bedtime and to end the measurement the next morning. In hospital, measurements were performed using BrainRT Shell + (OSG BVBA, Rumst, Belgium) and analyzed using BrainRT Shell + (version 1.0, Patch Pack 5, build 2570). Oronasal flow was measured with a thermal sensor. Breathing effort was measured through respiratory elastic belts at abdominal and chest level, heart rate using three electrocardiogram leads and SaO_2_ using an infant or pediatric oxygen sensor on a fingertip (OxiMax; Nellcor, Pleasanton, USA) applied to a pulse oxymeter (Xpod, Nonin Medical). Recordings of both devices were analyzed using the same methods as described below.

Due to the absence of electroencephalography, arousals were not recorded and therefore not used in the criteria. In some patients, one of the channels (SaO_2_, nasal flow or impedance) showed a technical failure. Measurements were excluded if either the impedance or the SaO_2_ was missing, because the purpose of the study was to detect central apneas. Mean pulse rate and mean respiratory rate were labeled as, respectively, tachycardia and tachypnea if they were >90th percentile for age [11].

### Scoring Respiratory Events

One observer (SvdB), blinded to the clinical characteristics of the patients, scored the sleep studies. All respiratory events were scored according to the American Academy of Sleep Medicine (AASM) criteria [12]. An *apnea* was defined as a drop in peak signal excursions of ≥90 %. *Central apnea* was scored if inspiratory effort was absent throughout the entire duration of the event and one of the following criteria were met: (1) the event lasted ≥20 s; (2) the event lasted for at least 2 breaths and was accompanied by an oxygen desaturation of ≥3 %. A central apnea following a sigh was scored only if it caused a desaturation ≥3 %. *Hypopnea* was defined as a reduction of ≥30 % of the pre-events baseline flow, lasted for at least 2 breaths and was accompanied by a desaturation of ≥3 %.

We calculated the *apnea*–*hypopnea index* (AHI) as the number of central apneas and hypopneas per hour of sleep. An AHI of ≥1 was defined as abnormal [13–17]. *Periodic breathing* was scored if ≥3 episodes of central apnea lasted >3 s separated by no more than 20 s of normal breathing.

### Statistical Analysis

All continuous variables are displayed as median (IQR), because of the low sample size in this study. Categorical variables are displayed as numbers and percentages. Difference between the median of two independent groups were assessed using the Mann–Whitney *U* test. Relationships between two non-normally distributed continuous variables were assessed using Spearman’s correlation. Statistical significance was defined as *p* < 0.05.

## Results

### Study Group

During the 3 years of the follow-up study, 58 of 79 eligible patients were willing to undergo polysomnography. Of these 58, eight patients were not measured, because they died or received a heart transplantation shortly after inclusion and before the polysomnography was performed; and in 13 patients the measurement failed, due to a lack of patient cooperation. As a result, 37 measurements were available.

The median age of the patients was 11.1 years. The median time since diagnosis of DCM was 3.6 years (range 0–15.6 years). Almost all patients (97 %) took angiotensin-converting enzyme inhibitors, 81 % took β-blockers and 70 % took diuretics as medical treatment for heart failure. The median LVEDD z-score was +4.7 and SF 19.4 %. (Table 1)

**Table 1:** Patient characteristics and clinical data within 3 months of the polysomnography

	All patients (*n* = 37)	No end point (*n* = 29)	Heart transplantation (*n* = 8)	*p* value
Male, *n* (%)	19 (51)			
Age (year)	11.1 (3.3–15.5)	8.7 (2.5–15.5)	12.3 (6.2–15.2)	NS
Etiology of DCM, *n* (%)				
Idiopathic	26 (70)			
Myocarditis	3 (8)			
Other^a^	8 (22)			
Time since diagnosis of DCM (year)	3.6 (1.6–7.6)			
Medication use, *n* (%)				
Diuretics	26 (70)	18 (62)	8 (100)	0.04
ACEi	36 (97)	29 (100)	7 (88)	NS
ß-blockers	30 (81)	22 (76)	8 (100)	NS
NYU PHFI	8 (5–11)	8 (4–10)	13 (10–14)	0.004
NT-pro BNP (pmol/L)	132 (79–480)	96 (50–195)	502 (417–776)	0.001
LVEDD z-score	+4.7 (3.3–6.9)	+4.1 (3.1–6.1)	+6.3 (3.7–9.3)	NS
SF (%)	19.4 (13.6–26.1)	19.7 (15.8–26.6)	12.2 (5.9–19.6)	0.01

Categorical variables are displayed as number (%), continuous variables are displayed as median (IQR)

*DCM* dilated cardiomyopathy, *ACEi* angiotensin-converting enzyme inhibitor, *NYU PHFI* New York University Pediatric Heart Failure Index, range 0–30, *NT*-*pro BNP* N-terminal B-type natriuretic peptide, *LVEDD* left ventricular end-diastolic dimension, *SF* shortening fraction

^a^ Category ‘other’ includes four patients with familial or genetic disease, three patients with prior use of anthracycline and one patient with vasculitis

The 34 patients in whom polysomnography was not performed (*n* = 21 not willing to undergo polysomnography and *n* = 13 measurement failure) were significantly younger (median age 3.3 years; *p* = 0.007) than the study group. LV dilation and function was not significantly different between groups (LVEDD z-score +5.8 [IQR 3.2−9.2], *p* = 0.3, and SF 16.8 % [IQR 11.9–19.8], *p* = 0.08).

### Sleep Study

Thirty-three patients (89 %) were measured at home with the ambulatory device, whereas four patients were measured in hospital. The median recording time was 513 min. Five recordings were shorter than 360 min (range 211–352). As we studied the prevalence of sleep-disordered breathing, we included these measurements in the analysis.

Of 37 patients, seven (19 %) had AHI ≥1 (range 1.2–4.5). These children were significantly younger than children with AHI <1 (median age 2.9 vs 12.3 year, *p* = 0.01). Three patients were younger than one year of age; all had an abnormal AHI (≥1) (Table 2).

**Table 2:** Polysomnography results

	All patients (*n* = 37)	No end point (*n* = 29)	Heart transplantation (*n* = 8)	*p* value
Total registration time (min)	513 (481–576)			
Resting heart rate (bpm)	78 (62–97)			
Tachycardia, *n* (%)	2 (5)			
Respiratory rate (/min)	21 (18–24)			
Tachypnea, n (%)	9 (24)			
Mean O_2_-saturation (%)	98 (97–98)			
Minimal O_2_-saturation (%)	91 (88–94)			
Mean O_2_-desaturation (%)	4.4 (3.9–5.2)			
AHI (/h)	0.2 (0.05–0.55)	0.2 (0–0.5)	0.44 (0.13–1.01)	NS
0–1/h, number of patients (%)	30 (81)	24 (83)	6 (75)	NS
1–5/h, number of patients (%)	7 (19)	5 (17)	2 (25)	
≥5/h, number of patients (%)	0 (0)			

Categorical variables are displayed as number (%), continuous variables are displayed as median (IQR). *AHI* apnea–hypopnea index, tachycardia and tachypnea defined as >90th percentile of the reference values [11]

**Fig. 1:**
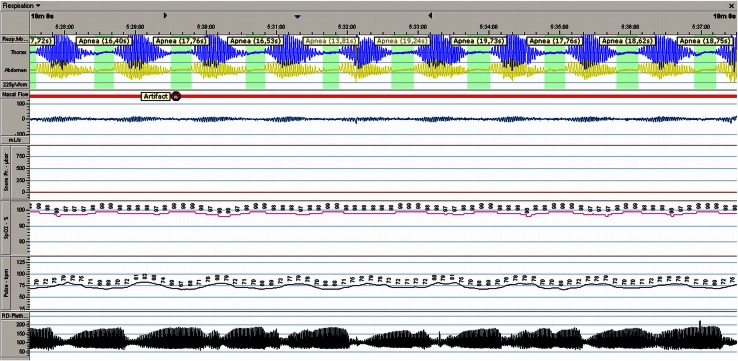
Recording of patient one (Table 3) showing a crescendo–decrescendo cycling pattern of the breathing amplitude with apneas

In three patients, clusters of apneas and hypopneas were noticed at the end of the night. The first patient (age 12 years) had a typical crescendo–decrescendo cycling pattern in breathing amplitude with apneas and hypopneas with a maximal episode duration of 58 min (Fig. 1). Only one apnea led to an oxygen desaturation, resulting in an AHI of only 0.1. The breathing pattern appeared as Cheyne–Stokes respiration and the duration of the episodes counted for 14.5 % of the total registration time (Table 3). The second patient (age 16 years) had several apneas >20 s with and without oxygen desaturations, reflected as an AHI of 2.6. The cycling pattern appeared as both Cheyne–Stokes and as periodic breathing and accounted for 27 % of the total registration time. The third patient (age 15 years) had a mild cycling breathing pattern without desaturations and a maximum duration of almost 17 min per episode (AHI 0). This breathing pattern appeared as periodic breathing and counted for 0.5 % of the total registration time.

**Table 3:** Three patients with patterns of hypopneas and apneas

	Patient 1	Patient 2	Patient 3
Age (year)	12.0	15.9	15.4
Number of apneas	34	95	1
AHI (/h of sleep)	0.1	2.6	0
Crescendo/decrescendo patterns			
Number	3	16	4
Minimal duration (mm:ss)	08:38	02:30	04:42
Maximal duration (mm:ss)	58:17	35:33	16:42
% of total sleep time	14.5	26.6	0.5

*AHI* apnea–hypopnea index

### Heart Failure Severity and Follow-up

During the measurement, tachycardia was present in two patients and tachypnea in nine patients.

The AHI was not correlated with the severity of heart failure symptoms, expressed as NYU PHFI, and also not with the severity of LV dysfunction and dilation (SF and LVEDD z-score), and not with the time since DCM diagnosis. During follow-up (median 2.0 years, IQR 1.3–3.2), eight patients underwent heart transplantation, no deaths occurred. The patients who underwent heart transplantation had more heart failure symptoms and worse LV function than those who survived without transplantation. AHI was not different between groups (Tables 1 and 2).

## Discussion

In this prospective observational study, we assessed the prevalence of CSA in children with heart failure and DCM. In 19 % of the children, we detected CSA, defined as AHI >1. We found no relation between the occurrence of CSA and the severity of heart failure in children. Three older patients showed episodes of a cycling pattern of crescendo–decrescendo changes in breathing amplitude with apneas and hypopneas, according to pediatric criteria defined as periodic breathing and mimicking Cheyne–Stokes respiration.

The prevalence of CSA in children with heart failure was lower than in adults with heart failure. We found an increased number of central sleep apneas and hypopneas in seven children (19 %), while reports in adults showed a prevalence of CSA around 35–40 % [1, 2, 4]. CSA in children was defined as AHI ≥1/h, while in adults CSA is defined as mild if AHI ≥5, moderate if AHI 15–29 and severe if AHI ≥30 [18]. Thus, as compared to adults with mean values reported around 30/h [1, 2, 4], the severity of CSA in children seemed relatively mild. Since the prevalence of CSA was low, we were not able to relate CSA to outcome.

Cheyne–Stokes respiration is a typical breathing abnormality, which is seen in adults with heart failure and associated with higher mortality rates [19]. In the current manual for scoring respiratory events in children, no rules for Cheyne–Stokes breathing are listed. However, scoring rules for periodic breathing are described for children and these mimic the rules for Cheyne–Stokes respiration [12]. Indeed, in three children a typical pattern of periodic breathing and Cheyne–Stokes respiration was detected. All were older children, respectively, 12, 15 and 16 years old. The two patients with the most severe manifestation of Cheyne–Stokes respiration had both severe DCM: both underwent transplantation, respectively, 14 and 22 months after the polysomnography; the third patient with mild periodic breathing is doing well on heart failure medication.

There may be several explanations why CSA in children with heart failure is less prevalent than in adults. One of the pathophysiological concepts of CSA is that the nocturnal fluid shift from legs to lungs stimulates pulmonary irritant chemoreceptors by pulmonary congestion, leading to hyperventilation and subsequently to a drop in PaCO_2_ below the apneic threshold. The amount of fluid that shifted from the legs was directly related to the amount of leg edema and sitting time, and inversely related to physical activity [20]. Leg edema has been associated with the presence of varicose veins and older age [21]. In contrast, fluid retention in children with heart failure is commonly associated with hepatomegaly, less often with ascites, but rarely with leg edema. These differences may be related to lower hydrostatic venous pressure in the legs and with more physical activity in children as compared to adults. Thus, the magnitude of the fluid shift may be smaller in children preventing the occurrence of CSA.

Another factor to take into consideration may be a difference in regulation of respiration in response to CO_2_. One of the mechanisms that results in a drop in PaCO_2_, leading to a central apnea, is hyperventilation initiated by an arousal [22]. Since the frequency of arousals increases with age [23, 24], children may have a lower number of arousals and subsequently a lower prevalence of CSA. Furthermore, in adults with heart failure an increased sensitivity to CO_2_ has been observed, and the sensitivity to CO_2_ has been positively correlated to the AHI [25]. In children, it is unknown whether such changes in the ventilatory response to CO_2_ exist and contribute to the occurrence of CSA. Interestingly, Cheyne–Stokes breathing pattern in our study was observed in the oldest children with severe heart failure. One may speculate that it occurs in those with severe heart failure and most resembling adults.

In our study, we reported the NYU PHFI to grade the severity of heart failure. We could not detect a correlation between NYU PHFI, LV function and dilation and the presence of CSA. The Cheyne–Stokes breathing pattern in two of eight children receiving a heart transplantation indicates that there might be a relation between the severity of heart failure and CSA, which needs further exploration.

This study has several limitations, which may have led to an underestimation or overestimation of the prevalence of CSA. Firstly, we recognize that we were unable to measure a substantial number of patients due to technical and practical problems. Although we used ambulatory devices, several parents and children were not willing to agree to undergo polysomnography. And probably, the use of ambulatory devices negatively influenced the success rate of the measurements. The children who were not measured were significantly younger, and their LV function was similar to children who were measured. Since the midterm prognosis of heart failure in young children is better than in older children [26], and we found more severe breathing abnormalities in older children, this may lower the impact of missing these children in this study. Secondly, eight children could not undergo a polysomnography because they either died or underwent heart transplantation before they could be studied. As in adults the prevalence increases with decreasing LV function, CSA may have been missed because we could not study this severely ill subgroup. Thirdly, in our cohort, three of the seven patients with AHI >1 were younger than 1 year of age. Particularly in young infants, central sleep apneas may be the result of immature breathing, rather than the result of heart failure [27]. The young age of the patients may have overestimated the prevalence. Fourthly, we included five recordings shorter than 360 min. Regardless of total registration duration, every central apnea was important to acknowledge calculating the prevalence. However, such short measurements without apneas did not guarantee that these patients were free from sleep apnea, and therefore may have led to an underestimation of the prevalence. Finally, as already mentioned, electroencephalography was not recorded. Therefore, the total sleep time may have been overestimated, and central apneas and hypopneas associated with arousals, rather than with desaturation, were not scored. This may have led to an underestimation of the AHI.

In the present study, only a relatively small patient group remained for final analysis. In order to increase the number of eligible patients in future research, we suggest to use in-hospital measurements on all newly diagnosed patients, since almost 80 % of the patients with DCM need hospital admission at diagnosis [26]. The use of in-hospital measurements may reduce the occurrence of technical failure. Furthermore, DCM symptoms may be worse during first admission, what may be therefore lead to a higher detection rate of CSA.

In conclusion, in this first prospective study to investigate the prevalence of central sleep apnea in children with moderate-to-severe heart failure, we found CSA in 19 % of the patients. In a small subset of older children with signs of severe heart failure, a Cheyne–Stokes respiration pattern was noticed, similar to that has been observed in adults. For the whole study group, no relation was found with the severity of heart failure.

All procedures performed in studies involving human participants were in accordance with the ethical standards of the institutional and/or national research committee and with the 1964 Helsinki declaration and its later amendments or comparable ethical standards.
